# Respiratory Virus Detection and Clinical Diagnosis in Children Attending Day Care

**DOI:** 10.1371/journal.pone.0159196

**Published:** 2016-07-19

**Authors:** Nina Moe, Bård Pedersen, Svein Arne Nordbø, Lars Høsøien Skanke, Sidsel Krokstad, Anastasios Smyrnaios, Henrik Døllner

**Affiliations:** 1 Department of Pediatrics, St. Olavs Hospital, Trondheim University Hospital, Trondheim, Norway; 2 Norwegian Institute for Nature Research, Trondheim, Norway; 3 Department of Medical Microbiology, St. Olavs Hospital, Trondheim University Hospital, Trondheim, Norway; 4 Department of Laboratory Medicine, Children’s and Women’s Health, Faculty of Medicine, Norwegian University of Science and Technology, Trondheim, Norway; Kliniken der Stadt Köln gGmbH, GERMANY

## Abstract

**Background:**

Respiratory viruses often have been studied in children with respiratory tract infection (RTI), but less knowledge exists about viruses in asymptomatic children. We have studied the occurrence of a broad panel of respiratory viruses in apparently healthy children attending day care, taking into account the influence of possible confounding factors, such as age, clinical signs of respiratory tract infection (RTI), location (day-care section) and season.

**Methods:**

We have studied 161 children in two day-care centers, each with separate sections for younger and older children, during four autumn and winter visits over a two-year period. A total of 355 clinical examinations were performed, and 343 nasopharyngeal samples (NPS) were analyzed by semi-quantitative, real-time, polymerase chain reaction (PCR) tests for 19 respiratory pathogens.

**Result:**

Forty-three percent of all NPS were PCR-positive for ≥ 1 of 13 virus species, with high species variation during visits. Rhinovirus 26% (88/343 NPS), enterovirus 12% (40/343) and parechovirus 9% (30/343) were detected in every visit, and the rates varied in relation to age, day-care section and season. Ten other viruses were detected in ≤ 3% of the NPS. Generally, viruses occurred together in the NPS. In 24% (79/331) of the clinical examinations with available NPS, the children had clear signs of RTI, while in 41% (135/331) they had mild signs, and in 35% (117/331) the children had no signs of RTI. Moreover, viruses were found in 70% (55/79) of children with clear signs of RTI, in 41% (55/135) with mild signs and in 30% (35/117) without any signs of RTI (p < 0.001).

**Conclusions:**

Positive PCR tests for respiratory viruses, particularly picornaviruses, were frequently detected in apparently healthy children attending day care. Virus detection rates were related to age, presence of clinical signs of RTI, location in day care and season.

## Introduction

The use of sensitive molecular tests such as polymerase chain reaction (PCR) has shown that several respiratory viruses frequently been detected in children who need hospitalization for respiratory tract infection (RTI) [1,2]. It has also been documented that children hospitalized with RTI often have multiple viruses, and that asymptomatic hospital controls may frequently be positive for respiratory viruses [3–6]. Outside the hospital setting, evidence exists for the presence of both well-known and recently detected viruses, such as respiratory syncytial virus (RSV), human rhinovirus (HRV), human metapneumovirus (hMPV) and human bocavirus (HBoV), in children with RTI [7,8]. However, it is more surprising that even asymptomatic children outside hospitals may harbor viruses in their airways, as has been recently shown [9–11]. We aim to study this phenomenon further, and describe the occurrence of a broad panel of respiratory pathogens in healthy children. Since nearly all Norwegian children attend day care on a daily basis, we have studied a group of apparently healthy children attending day care, taking into account the influence of possible confounding factors such as age, clinical signs of RTI, location in day care and season.

## Materials and Methods

### Study Population

**Fig 1:**
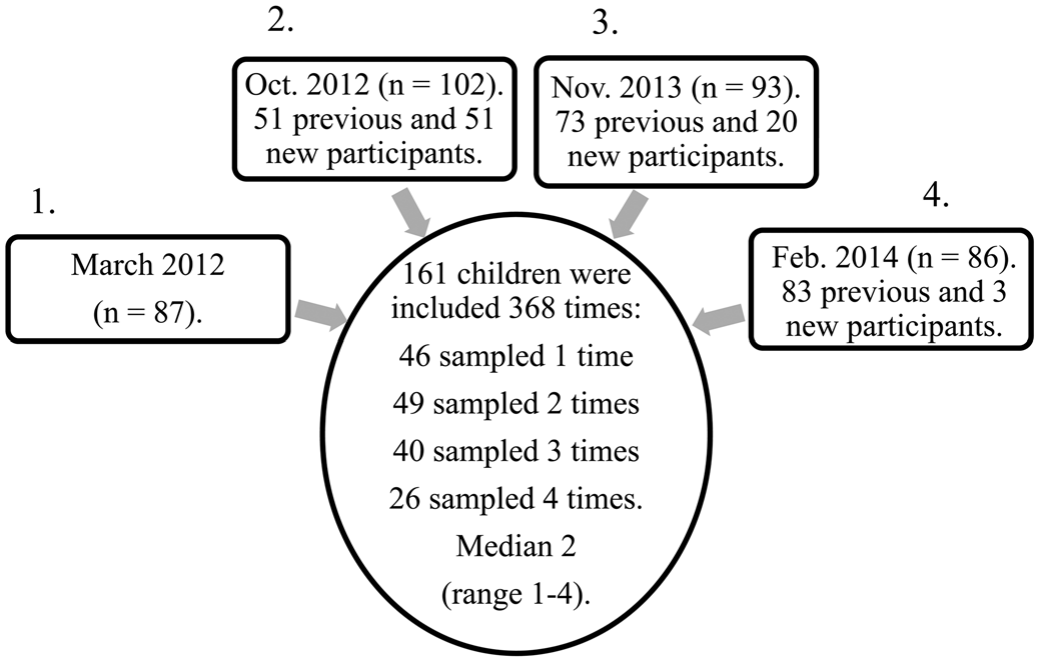
Design of the study. Number of included children during each of four study visits and the number of children being sampled one, two, three or four times.

The study was performed during four visits between March 2012 and February 2014 in two day-care centers in the city of Trondheim, Norway, with 95% of all toddlers and preschool children in Trondheim attended day care during the study period (Statistics Norway 2014). Norwegian children start school at the age of six, and the children included were between the ages of 1–6.3 years. The number of children in the two day-care centers varied from 110 to 132 at each visit. The children were organized into five or six sections (the number differed during the two years), with 6–12 of the youngest children per section, aged 1–3.8 years, and four sections for the oldest with 16–18 children per section, aged 2.8–6.3 years. In total, 161 children participated in the study one or more times (median two times, range one to four), which resulted in 368 out of 484 possible (76.0%) inclusions (Fig 1). The majority of included children was both sampled by a nasopharyngeal sample (NPS) and underwent clinical examination, although some resisted the collection of NPS or clinical examination after inclusion. With the exception of one, all children usually stayed 41 hours per week in the day-care center. The inclusion criterion was informed written consent from parents or guardians on behalf of the children for each study visit. Each child could be included only once during each study visit. The exclusion criterion was previous nasal bleeding. At each inclusion, the parents answered a form of baseline demographics, household characteristics and medical history. One of four pediatricians conducted a standardized clinical examination of each child during daytime in the day-care area. The pediatricians classified the children into three groups based on clinical findings: 1. No RTI with normal findings, 2. Mild RTI with discrete signs of rhinitis, pharyngitis, simplex media otitis or secretory media otitis, and 3. Clear RTI with significant signs of rhino-pharyngitis, tonsillitis, purulent media otitis or auscultatory findings from the lower airways. The study was approved by the Regional Committee for Medical and Health Research Ethics, Mid-Norway, Norway (no. 2011/2246).

### Sampling and Microbiologic Analyses

Nasopharyngeal samples were obtained by flocked swabs (Copan Italia SpA^®^) and placed immediately into a 3 ml transport medium (UTM-RT, Copan Italia SpA^®^). Samples were analyzed at the Department of Medical Microbiology, St. Olavs Hospital, Trondheim University Hospital, Trondheim, Norway. In total, 361 NPS were collected, though some samples of poor quality (n = 18) were excluded. The NPS were analyzed with semi-quantitative, real-time PCR for 19 respiratory pathogens including human adenovirus (HAdV), HBoV, human coronavirus (HCoV) OC43, 229E, NL63, human enterovirus (HEV), human parechovirus (HPeV), hMPV, influenza A virus, influenza B virus, parainfluenza virus (PIV) types 1–4, RSV, HRV, *Bordetella pertussis*, *Chlamydophila pneumoniae* and *Mycoplasma pneumoniae*. The PCRs were in-house, real-time assays with TaqMan probes [12]. The amount of virus in each sample was recorded semi-quantitatively, and based on the cycle threshold value (Ct-value). Ct-values above 40 were regarded as virus-negative.

### Statistical Analyses

The χ^2^ or a Fisher’s Exact Test were used to compare differences in proportions, and continuous but not normally distributed data were analyzed by use of a Kruskal-Wallis test. The Monte Carlo simulation test described by Hope was used to test whether respiratory pathogens occurred independently of each other among children, using the algorithm by Patefield [13,14]. The test compared the observed distribution of the number of pathogens in a nasopharyngeal sample, with a distribution based on the assumption that pathogens occurred independently of each other and conditional on their observed frequencies. The test was based on 2,000 simulations of the null hypothesis. Following the rejection of the null hypothesis (see Results), the same approach was subsequently used to test whether the distribution of pathogens among day-care sections and sampling times could account for the general tendency of respiratory pathogens to occur together in NPS. In addition, in the latter test, the null distribution was conditional on the distribution of pathogens among day-care sections and sampling times. Hope’s test was further used to test in pairs whether the three most common pathogens, HEV, HPeV and HRV, occurred independently of each other. The sequential Bonferroni method was also used to control the familywise Type I error rate in these three tests [15]. The occurrence in NPS of the same three respiratory pathogens was analyzed in an explorative manner using generalized linear mixed-effect models with logit link functions [16]. Day-care sections and sampling times (seasons) were included in the logistic models as random explanatory variables, while the children’s age in months and the occurrence of other viruses (coded as a binary variable) were included as fixed variables. The “top-down” approach recommended by Diggle et al. was followed, in which the random part of models was first determined based on the “beyond optimal model”, before obtaining the minimal adequate model by selecting among the candidate’s fixed parts [17,18]. Model selection was based on the Akaike information criterion (AIC) [19]. The same approach was followed in order to study whether clinical findings were related to the occurrence of HRV, which was the virus most frequently found in the NPS. Day-care sections and sampling times (seasons) were again included as random explanatory variables, whereas the occurrence of HRV and children’s age were included as fixed variables. The response variable was the occurrence of clear findings of RTI coded as a binary variable, with mild and no RTI findings as the reference category. Moreover, statistically significant values were defined as p<0.05 (two-sided), and IBM SPSS Statistics 22 and R version 3.2.2 were used in the statistical analyses [20]. The R-package lme4 was used in the GLMM-modelling [21].

## Results

### Viral Findings

**Fig 2:**
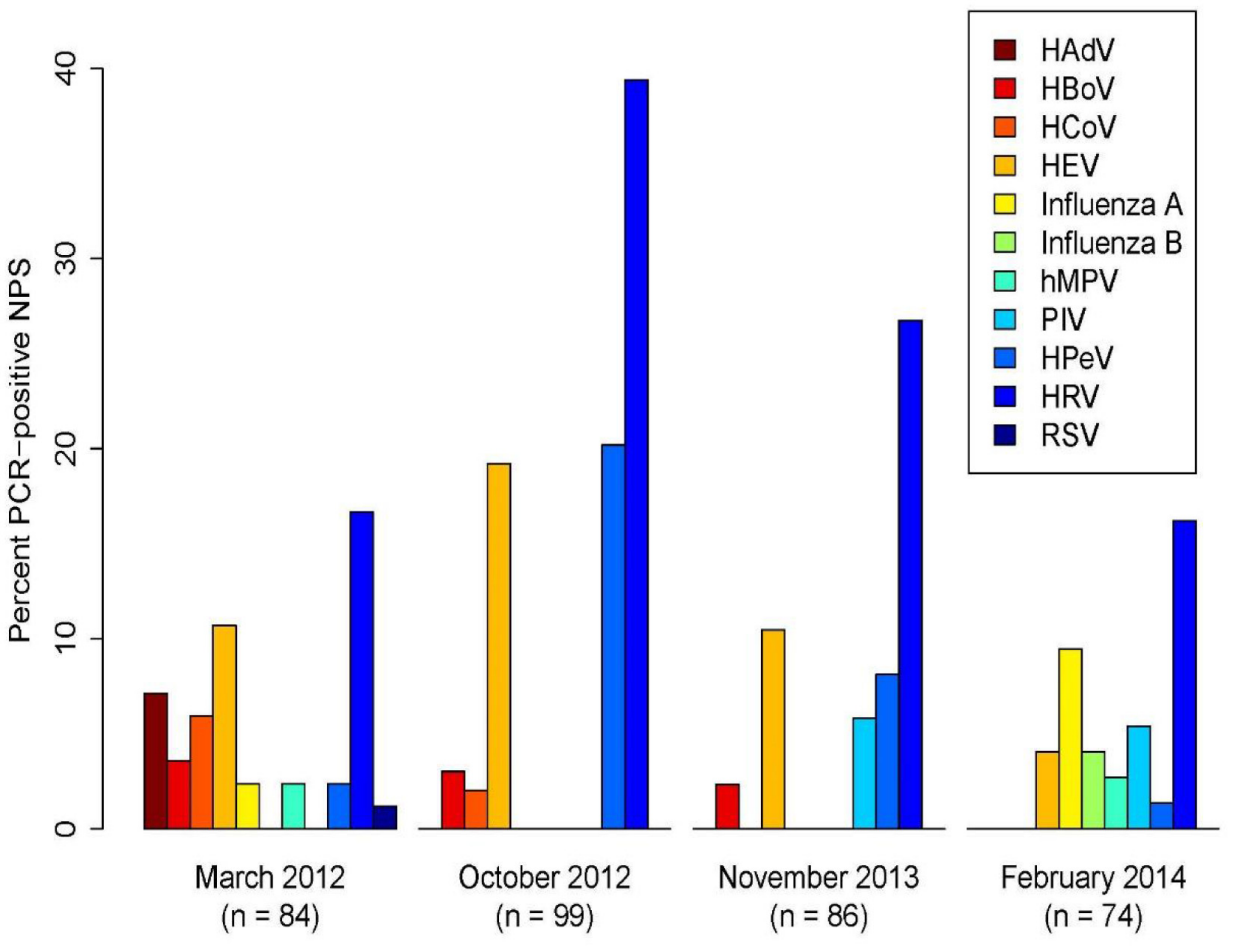
Viral findings at each study visit. Percent nasopharyngeal samples that were positive for each of 11 virus types (genotypes of HCoV and PIV not shown). Nasopharyngeal samples were collected at four different sampling times.

NPS were collected in 343 out of the 368 inclusions (93.2%). Overall, 149 (43%) of the samples were PCR-positive for virus, varying from 34% (25/74) to 56% (55/99) at each study visit (Table A in S1 File). There was a large variation in pathogen detections during the four visits (Fig 2), and only HEV, HPeV, and HRV were detected at all visits. HRV was the most frequent, detected in 88 out of 343 samples (26%), varying from 16% (12/74) to 39% (39/99) at each visit, while HEV was detected in 12% (40/343) and HPeV in 9% (30/343). Ten other viruses were each detected in ≤ 3%, including HAdV (n = 6) and HBoV (n = 8), and none was positive for HCoV-OC43, PIV 2 and 3, *Bordetella pertussis*, *Chlamydophila pneumoniae* or *Mycoplasma pneumoniae*.

**Fig 3:**
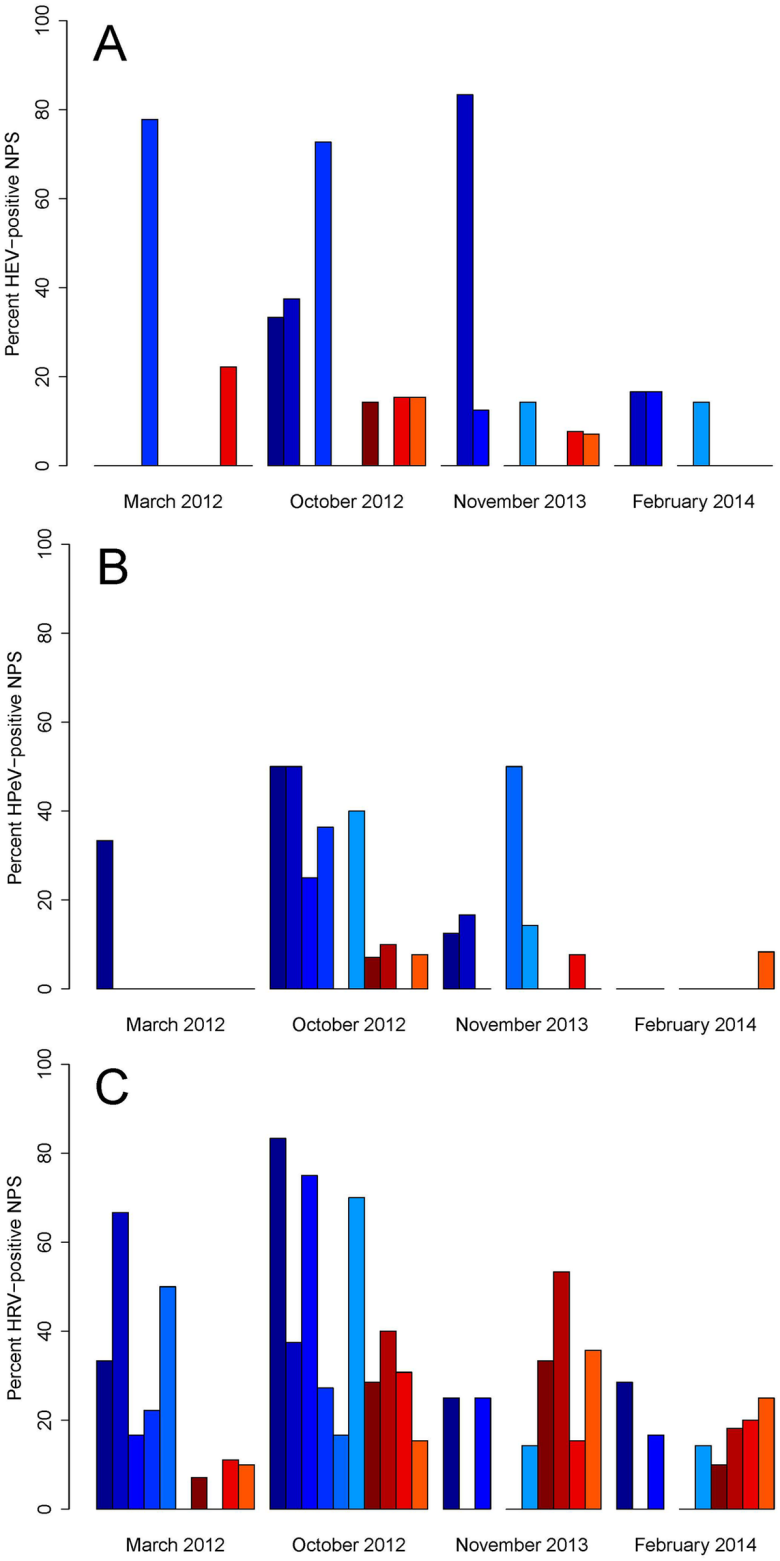
Occurrence of picornaviruses. Percent positive nasopharyngeal samples (NPS) for human enterovirus (HEV) (A), human parechovirus (HPeV) (B) and human rhinovirus (HRV) (C) at four different study visits (sampling times) and in each of 10 day-care sections, six young children sections (blue colors) and four older children sections (red colors). One of the young children sections was not sampled in November 2013 and February 2014.

One virus was detected in 31% (106/343) of the NPS, and two or more viruses were detected in 12% (43/343) (Table A in S1 File). NPS with multiple viruses (≥ 2 viruses) were more frequent than expected if the viruses were randomly and independently distributed among NPS, while single virus samples were less frequent than expected (χ^2^ = 21.6, p = 0.0045). Thus, there was a general tendency that viruses occurred together in NPS, although this tendency was not due to the uneven occurrence of viruses among day-care sections and sampling times (Figs 2 and 3, χ^2^ = 30.2, p = 0.0020). The co-detection of other viruses appeared in 30 out of 88 HRV-positive samples (34%). The corresponding figures for HEV and HPeV were even higher (23 co-detections out of 40 HEV-positive samples (58%) and 20 co-detections out of 30 HPeV-positive samples (67%)) (Table A in S1 File). HPeV was positively associated with both HEV (χ^2^ = 10.7, p = 0.0020) and HRV (χ^2^ = 5.4, p = 0.021), while HEV and HRV did not occur more often together than expected by chance (χ^2^ = 1.1, p = 0.34). In addition, several of the less frequent virus types, e.g. HBoV, hMPV and PIV, seemed to be positively associated with other viruses (Table B in S1 File).

One or more viruses were detected in 55% (83/152) of the NPS from sections with young children, compared to 35% (66/191) of the samples from older children (p < 0.001). The following virus species were only detected in sections with young children: RSV, PIV-1, hMPV, and HCoV-NL63.

According to the GLMM analysis, the occurrence of HEV-positive NPS varied randomly among combinations of sections and sampling times (Fig 3A); this means that the occurrence of HEV varied from zero to approximately 80% between sections, but it was not the same sections that had a low or high prevalence each time. The probability of HEV-positive NPS decreased with an increasing age of children (z-test: z = -2.4, p = 0.016), and increased with the presence of other viruses (z-test: z = 2.8, p = 0.005). The median age of the HEV positives was 28 months (interquartile range (IQR) 19.3–34.0). Nearly similar results were obtained when modelling the occurrence of HPeV. It varied randomly among sampling times (Fig 3B), decreasing with the increasing age of children (z-test: z = -4.1, p = 0.001), and increasing marginally with the presence of other viruses (z-test: z = 1.7, p = 0.090). The median age of the HPeV positives was 22.5 months (IQR 17.0–30.3). The occurrence of HRV also varied randomly among combinations of sections and sampling times (Fig 3C). There was also a positive effect of the presence of other viruses (z-test: z = 2.0, p = 0.044); however, the presence of HRV was not related to the age of the children (likelihood ratio test: χ^2^ = 0.6, df = 1, p = 0.428). Compared to the HEV and HPeV positives, the HRV positives had a higher median age of 35.5 months (IQR 21.0–54.0).

### Clinical Findings

In 355 of the 368 inclusions, a clinical examination was performed (96%). NPS were collected from 331 of the examined children, among whom 24% (79/331) had clear findings of a RTI, 41% (135/331) had mild findings and 35% (117/331) had normal findings (Table 1). The baseline characteristics of the children (n = 331) showed that children with a clear RTI were younger and more frequently reported to have more than four upper RTIs per year, compared to those with a mild RTI and no RTI findings (Table 1).

**Table 1:** Baseline characteristics of children at 331 inclusions in the study.

Characteristics	No RTI (n = 117)	Mild RTI (n = 135)	Clear RTI (n = 79)	*P*
**Age, month, median (IQR)**	51 (41–62)	35 (25–53)	30 (18–47)	<0.001
**0–2 years**	8 (7)	30 (22)	28 (35)	<0.001**
**2–4 years**	41 (35)	60 (44)	32 (41)	
**4–6.2 years**	68 (58)	45 (33)	19 (24)	
**Male gender**	73 (62)	75 (56)	53 (67)	0.224
**Young children section**	20 (17)	71 (53)	51 (65)	<0.001***
**Older children section**	97 (83)	64 (47)	28 (35)	
Parental reports received*	92 (79)	114 (84)	64 (81)	0.489
**Premature < 36 GA**	11 (12)	8 (7)	4 (6)	0.342
**Siblings ≥ 1**	79 (86)	83 (73)	42 (66)	0.010
**Pets**	24 (26)	25 (22)	12 (19)	0.546
**≥ 1 parents smoking**	9 (10)	11 (10)	7 (11)	0.959
**Vaccination**	92 (100)	114 (100)	63 (98)	NA
**Antibiotic treatment last 6 months**	4 (4)	16 (14)	7 (11)	0.068
**>4 upper RTI per year**	13 (14)	23 (20)	20 (31)	0.034
**Allergy**	10 (11)	10 (9)	8 (13)	0.722
**Asthma**	7 (8)	8 (7)	5 (8)	0.977
**Eczema**	12 (13)	13 (11)	12 (19)	0.382
**Epilepsy**	0	1 (1)	1 (2)	NA
**Heart disease**	0	0	1 (2)	NA
**Other chronic diseases**	1 (1)	1 (1)	0	NA

Data presented as absolute numbers and percent in parenthesis, except from age in months and IQR (interquartile range).

*The number of parental reports received are basis (100%) when calculating percent for all variables except age, gender, Young children section and Older children section. P-values calculated with χ² test, except Kruskal-Wallis test for comparing median age.

**Comparing all three age categories.

***Comparing Young children section with Older children section.

GA, gestational age. NA, not applicable. RTI, respiratory tract infection.

### Association between Viral and Clinical Findings

Seventy percent (55/79) of the children with clear signs of a RTI had one or more viruses in the NPS, compared to 41% (55/135) in those with mild findings and 30% (35/117) in those without a RTI (p < 0.001) (Table 2). Among the children with a clear RTI and positive NPS, 45% (25/55) were younger than 2 years old (Table 2). HRV was the most frequently detected virus in all clinical groups, varying from 41% (32/79) in the clear RTI group, to 24% (32/135) in the mild group, and 18% (21/117) in children without a RTI (p = 0.001) (Table 2). The Ct-values for HRV in NPS were not significantly different between the groups (data not shown). The minimal adequate model from the GLMM analysis of the occurrence of clear findings of a RTI included positive effects of the occurrence of HRV (z-test: z = 3.0, p = 0.002, and Table 2) and negative effects of children’s increasing age (z-test: z = -3.2, p = 0.001), together with random effects of combinations of day-care sections and sampling times. HEV and HPeV were also detected in all three groups, and most frequently in those with a clear RTI (p = 0.003 and 0.005, respectively, Table 2). GLMM analyses of the occurrence of a clear RTI with the presence of HEV or HPeV were inconclusive (data not shown). A few children (n = 12) had influenza viruses A/B, among whom nine had clear signs of a RTI (Table 2). Only 14 children had hMPV (n = 4), RSV (n = 1) or PIV (n = 9), where 11 had mild or clear signs of a RTI. Multiple viruses were detected in 27% (21/79) of NPS from children with a clear RTI, compared to 9% (12/135) in those with mild and 9% (10/117) with normal findings (p < 0.001) (Table 2 and Table C in S1 File).

**Table 2:** Viral Findings in 331 inclusions in which children had no, mild or clear findings of a respiratory tract infection (RTI).

Viral findings	Total	No RTI (n = 117)	Mild RTI (n = 135)	Clear RTI (n = 79)	No vs Mild RTI *p*-value	Mild vs Clear RTI *p*-value	No vs Clear RTI *p*-value	All groups *p*-value
**Virus negative**	186 (56)	82 (70)	80 (59)	24 (30)	0.157*	<0.001*	<0.001*	<0.001*
**SV positive**	102 (31)	25 (21)	43 (32)	34 (43)				
**MV positive**	43 (13)	10 (9)	12 (9)	21 (27)				
**Positive any virus**	145 (44)	35 (30)	55 (41)	55 (70)	0.074	<0.001	<0.001	<0.001
**Age 0–2 years**	43 (30)	4 (11)	14 (26)	25 (45)	0.016**	0.063**	0.001**	0.001**
**Age 2–4 years**	53 (36)	11 (31)	26 (47)	16 (29)				
**Age 4–6.2 years**	49 (34)	20 (57)	15 (27)	14 (25)				
**HEV**	40 (12)	9 (8)	13 (10)	18 (23)	0.587	0.008	0.003	0.003
**HPeV**	29 (9)	6 (5)	9 (7)	14 (18)	0.607	0.012	0.004	0.005
**HRV**	85 (26)	21 (18)	32 (24)	32 (41)	0.264	0.010	<0.001	0.001
**HAdV**	6 (2)	0 (0)	5 (4)	1 (1)	0.063	0.417	0.403	NA
**HBoV**	8 (2)	3 (3)	0 (0)	5 (6)	0.099	0.006	0.272	NA
**HCoV**	7 (2)	3 (3)	3 (2)	1 (1)	1.0	1.0	0.649	NA
**Influenza A**	9 (3)	2 (2)	1 (1)	6 (8)	0.598	0.011	0.063	NA
**Influenza B**	3 (1)	0 (0)	0 (0)	3 (4)	NA	0.049	0.064	NA
**hMPV**	4 (1)	0 (0)	3 (2)	1 (1)	0.251	1.0	0.403	NA
**PIV**	9 (3)	3 (3)	2 (1)	4 (5)	0.666	0.196	0.443	NA
**RSV**	1 (0)	0 (0)	1 (1)	0 (0)	1.0	1.0	NA	NA

Data presented as absolute numbers and percentage in parenthesis. P-values using χ² test or Fischer’s Exact Test. RTI, respiratory tract infection. SV, single virus. MV, multiple viruses, with ≥2 viruses. NA, not applicable.

*Comparing Virus negative, SV positive and MV positive.

**Comparing all three age categories.

### Parental Reported Symptoms

Based on information collected from the parents, 84% (54/64), 65% (74/113) and 45% (40/89) of the children with clear, mild or no clinical signs of RTI had respiratory symptoms at the examination time or two weeks prior. Among the 55% (49/89) without reported symptoms and with normal findings, still 24% (12/49) had one or more viruses: HCoV-229E (n = 1), HEV (n = 2), HPeV (n = 3) and HRV (n = 8).

## Discussion

We detected one or more respiratory viruses in four out of 10 Norwegian children attending day care. All children participated in daily activities, but nevertheless one-fourth had clear signs of an ongoing RTI by clinical examination, and approximately four out of ten had milder signs of RTI. Although those with clear signs had the highest virus detection rate (70%), one-third was still virus positive and without any clinical signs. Hence, our findings indicate that apparently healthy day-care children may harbor respiratory viruses and have clinical signs of an upper RTI, and even children without clinical signs may be virus positive. It is well-known that young children frequently have symptomatic RTIs, so it is not surprising that children sometimes may also have a RTI with few symptoms when they attend day care [22,23].

Picornaviruses were the most frequently detected viruses during all four sampling times, whereas RSV, influenza virus and other significant pathogens were identified in less than one-fifth of the picornaviruses, and primarily in those with clear RTIs. One out of four visits to the day-care centers occurred during a RSV epidemic, which might explain that only one child had RSV. However, it may be possible that RSV more often causes severe disease and sick leave from day care. Rhinovirus appeared most frequently, but enterovirus and parechovirus were also common. Combinations of day-care sections (younger or older children) and sampling times (seasons) were the most important factors in determining the occurrence of picornaviruses. At any given sampling time, there was a large variation in the frequencies of the three picornaviruses among the various sections, and for most of the sections, there was a large variation at different sampling times. These observations may be relate to the fact that most respiratory viruses are epidemic and easily spread among children who are cared for in separate sections [24]. Indeed, this phenomenon was most common in the sections for the youngest children, who—in particular—are known to challenge good hygiene in day-care centers.

There was a general tendency that viruses occurred together, independent of the influence of sections and sampling times. For instance, the detection of HPeV was associated with both the presence of HEV and HRV, whereas HEV and HRV were related to other viruses, but not to each other. Similarly, others reported that some virus combinations may appear more frequently than others in both children with and without RTI, and that co-infections with viruses may not be random in children with RTI [25–28]. Martin et al. showed that during the progress of a RTI in children, more respiratory viruses may appear [26]. Our data revealed that children with a clear RTI often had frequent upper RTIs during the six months prior to the inclusion in the study, and therefore might have a higher risk of being PCR positive for more than one viruses simultaneously, which is due to possible long-term viral excretion after clinical recovery.

HRV was detected at every sampling time, and was the most common virus. HRV occurred in both the sections of younger and older children, and varied randomly among combinations of sections and sampling times. Children with HRV-positive NPS had increased probability of a clear RTI. Consequently, in this study, HRV was the likely cause of many RTIs in children outside of a hospital, as has been shown by others [22,26,29–31]. However, we also detected HRV in nearly one-fifth of the children without clinical findings of a RTI, while others have detected HRV in asymptomatic children, which is more difficult to explain [9–11,30]. Peltola et al. examined various HRV strains and found that a minor fraction of HRV infections in children may be asymptomatic, and it has also been suggested that HRV PCR tests may persist as positive up to several weeks after clinical recovery [32,33]. Hence, our HRV detection in children without clinical findings can be a result of the carriage of virus after the recovery of symptoms or a newly acquired asymptomatic infection. We found that several children with HRV and clear signs of RTI attended day care and were apparently healthy, which could suggest that HRV in other cases may also cause very mild changes that are hard to detect at all. Recent data have shown that HRV-positive children with and without symptoms developed different systemic immune responses, which support that HRV detection may not always indicate symptomatic HRV infection [34].

HPeV in children has previously been examined only in a few studies, with low detection rates from 1.6% to 2.1% in hospitalized children with RTI, but in a group of asymptomatic young children van den Berg recently detected HPeV in 9% [27,35,36]. Serological studies have documented that most Finnish children may be infected with HPeV1 (83%) and HPeV2 (91%) before the age of five years [37]. HPeV3 is strongly related to sepsis-like disease and encephalitis, though not RTI, in infants [38]. In the present study, HPeV and HEV were often detected in the same children, attending sections for young children. This co-variation and possible confounding eliminated our possibility to prove that HPeV and HEV were actually related to RTI among the youngest children.

We only detected a few HBoV-positive samples in children with a clear RTI, as well in children who had no signs of RTI, and adenovirus appeared mostly in children with a mild RTI. Recent evidence support that HBoV may cause acute RTI, and adenovirus is a well-known cause of RTI [3,39]. However, it has also been shown that these viruses in particular may sometimes be detected for a long time in the airways, either due to prolonged excretion or due to the re-activation of a latent infection, and all three mechanisms may explain our findings [40,41].

To describe complex microbiology, we collected seasonal samples in both the fall and winter periods in two consecutive years. The day-care section was also considered, and turned out to be an important predictor of virus occurrence. Nasopharyngeal sampling is unpleasant and challenging to perform in apparently healthy children outside health institutions. However, we managed to collect NPS from more than 90% of the inclusions. Ideally, more samples from each child, using a stricter longitudinal design, might have had advantages over the present cross-sectional approach, but in real-life frequent sampling was not possible to attain. A major strength of the study is that pediatricians clinically examined all children, and their findings were used in the classification. Most studies on respiratory viruses and RTIs in day-care settings have relied on parental information of children’s symptoms. Nonetheless, we found a poor correlation between symptoms and clinical signs. Others have similarly shown that symptoms are not entirely accurate in predicting an upper RTI in children [42]. On the other hand, the clinical entities of a mild and clear RTI, which were used in our classification, have not been validated. Each study visit was performed three-12 months apart, which is a long time from an epidemic and clinical perspective and, therefore, the analyses were not adjusted for repetitive data.

In conclusion, this study showed that 43% of apparently healthy children attending day care had one or more viruses in NPS, varying from 30% in children with no clinical findings to 70% in those with clear findings of a RTI. Picornaviruses were most frequently detected. Lastly, the viral occurrences were related to age, clinical signs of RTI, location in day care and sampling times (seasons).

## Supporting Information

S1 File **Table A**. Number virus in nasopharyngeal samples collected at four study visits, and number of nasopharyngeal samples with multiple virus (MV). **Table B**. Virus combinations in 43 nasopharyngeal samples with multiple virus. **Table C**. Virus combinations in 43 nasopharyngeal samples with multiple viruses, in children with clear, mild or no respiratory tract infection (RTI).
(DOCX)
