# Supplementary material for: Respiratory Virus Detection and Clinical Diagnosis in Children Attending Day Care
Source: PLoS One. 2016 Jul 19;11(7):e0159196. doi: 10.1371/journal.pone.0159196 (PMC4951077; doi:10.1371/journal.pone.0159196)
Supplement: S1 File — Table A. Number virus in nasopharyngeal samples collected at four study visits, and number of nasopharyngeal samples with multiple virus (MV). Table B. Virus combinations in 43 nasopharyngeal samples with multiple virus. Table C. Virus combinations in 43 nasopharyngeal samples with multiple viruses, in children with clear, mild or no respiratory tract infection (RTI). (DOCX) [file pone.0159196.s001.docx]

**S1 file**

**Table A. Number of viruses in nasopharyngeal samples collected at four study visits, and number of nasopharyngeal samples with multiple viruses (MV).**

|  |  |  |  |  |  |  |
| --- | --- | --- | --- | --- | --- | --- |
| NPS result | **March 12**  **n = 84** | **Oct. 12**  **n = 99** | **Nov. 13**  **n = 86** | **Feb. 14**  **n = 74** | **Total**  **n = 343** | **MV** |
| Virus negative | 50 (60) | 44 (44) | 51 (59) | 49 (66) | 194 (57) |  |
| Positive any virus | 34 (40) | 55 (56) | 35 (41) | 25 (34) | 149 (43) |  |
| Single virus | 26 (31) | 33 (33) | 27 (31) | 20 (27) | 106 (31) |  |
| Multiple viruses | 8 (9) | 22 (22) | 8 (9) | 5 (7) | 43 (12) |  |
| 2 viruses | 6 (7) | 17 (17) | 6 (7) | 3 (4) | 32 (9) |  |
| ≥ 3 viruses | 2 (2) | 5 (5) | 2 (2) | 2 (3) | 11 (3) |  |
| HAdV | 6 (7) | 0 (0) | 0 (0) | 0 (0) | 6 (2) | 2 |
| HBoV | 3 (4) | 3 (3) | 2 (2) | 0 (0) | 8 (2) | 8 |
| HCoV: | 5 (6) | 2 (2) | 0 (0) | 0 (0) | 7 (2) | 3 |
| HCoV-229E | 5 (6) | 0 (0) | 0 (0) | 0 (0) | 5 (1) |  |
| HCoV-NL63 | 0 (0) | 2 (2) | 0 (0) | 0 (0) | 2 (1) |  |
| HCoV-OC43 | 0 (0) | 0 (0) | 0 (0) | 0 (0) | 0 (0) |  |
| HEV | 9 (11) | 19 (19) | 9 (10) | 3 (4) | 40 (12) | 23 |
| Influenza A virus | 2 (2) | 0 (0) | 0 (0) | 7 (9) | 9 (3) | 1 |
| Influenza B virus | 0 (0) | 0 (0) | 0 (0) | 3 (4) | 3 (1) | 1 |
| hMPV | 2 (2) | 0 (0) | 0 (0) | 2 (3) | 4 (1) | 2 |
| HPeV | 2 (2) | 20 (20) | 7 (8) | 1 (1) | 30 (9) | 20 |
| PIV: | 0 (0) | 0 (0) | 5 (6) | 4 (5) | 9 (3) | 8 |
| PIV 1 | 0 (0) | 0 (0) | 1 (1) | 0 (0) | 1 (0) |  |
| PIV 2-3 | 0 (0) | 0 (0) | 0 (0) | 0 (0) | 0 (0) |  |
| PIV 4 | 0 (0) | 0 (0) | 4 (5) | 4 (5) | 8 (2) |  |
| HRV | 14 (17) | 39 (39) | 23 (27) | 12 (16) | 88 (26) | 30 |
| RSV | 1 (1) | 0 (0) | 0 (0) | 0 (0) | 1 (0) | 1 |

Data presented as absolute numbers and percent in parenthesis, except from absolute numbers of MV.

MV, multiple viruses with ≥ 2 viruses in the nasopharyngeal sample.

**Table B.** **Virus combinations in 43 nasopharyngeal samples with multiple viruses.**

|  |  |  |  |  |  |  |  |  |  |  |  |
| --- | --- | --- | --- | --- | --- | --- | --- | --- | --- | --- | --- |
|  | **HAdV**  **n=2** | **HBoV**  **n=8** | **HCoV**  **n=3** | **HEV**  **n=23** | **Infl A**  **n=1** | **Infl B**  **n=1** | **hMPV**  **n=2** | **HPeV**  **n=20** | **PIV**  **n=8** | **HRV**  **n=30** | **RSV**  **n=1** |
| HAdV | - |  |  |  |  |  |  |  |  | 2 | 1 |
| HBoV |  | - | 1 | 3 |  |  |  | 3 | 1 | 6 |  |
| HCoV |  | 1 | - |  |  |  | 1 | 1 |  | 1 |  |
| HEV |  | 3 |  | - | 1 |  |  | 9 | 4 | 13 |  |
| Infl A |  |  |  | 1 | - |  |  |  |  |  |  |
| Infl B |  |  |  |  |  | - |  |  | 1 | 1 |  |
| hMPV |  |  | 1 |  |  |  | - |  |  | 1 |  |
| HPeV |  | 3 | 1 | 9 |  |  |  | - | 2 | 13 |  |
| PIV |  | 1 |  | 4 |  | 1 |  | 2 | - | 5 |  |
| HRV | 2 | 6 | 1 | 13 |  | 1 | 1 | 13 | 5 | - | 1 |
| RSV | 1 |  |  |  |  |  |  |  |  | 1 | - |

Infl A, influenza A virus. Infl B, influenza B virus. Multiple viruses with ≥ 2 viruses in the nasopharyngeal sample.

**Table C. Virus combinations in 43 nasopharyngeal samples with multiple viruses in**

**children with clear, mild or no respiratory tract infection (RTI).**

|  |  |  |  |  |  |  |  |
| --- | --- | --- | --- | --- | --- | --- | --- |
|  | **2 viruses** | **N** | **3 viruses** | **N** | **4 viruses** | **N** | **Total** |
| Clear RTI | All | 13 | All | 7 | All | 1 | 21 |
|  | HEV, HRV | 5 | HBoV, HEV, HRV | 1 | HBoV, HEV, HPeV, HRV | 1 |  |
|  | HEV, HPeV | 2 | HBoV, HPeV, HRV | 1 |  |  |  |
|  | HEV, infl A | 1 | HCoV, HPeV, HRV | 1 |  |  |  |
|  | HPeV, HRV | 2 | HEV, HPeV, HRV | 2 |  |  |  |
|  | HEV, HBoV | 1 | HEV, HRV, PIV | 1 |  |  |  |
|  | HBoV, HRV | 1 | HRV, infl B, PIV | 1 |  |  |  |
|  | HPeV, PIV | 1 |  |  |  |  |  |
| Mild RTI | All | 10 | All | 2 | All | 0 | 12 |
|  | HCoV, hMPV | 1 | HAdV, HRV,RSV | 1 |  |  |  |
|  | HPeV, HRV | 2 | HEV, HRV, PIV | 1 |  |  |  |
|  | HEV, HRV | 1 |  |  |  |  |  |
|  | HEV, HPeV | 3 |  |  |  |  |  |
|  | hMPV, HRV | 1 |  |  |  |  |  |
|  | HEV, PIV | 1 |  |  |  |  |  |
|  | HAdV, HRV | 1 |  |  |  |  |  |
| No RTI | All | 9 | All | 0 | All | 1 | 10 |
|  | HBoV, HCoV | 1 |  |  | HBoV, HPeV, HRV, PIV | 1 |  |
|  | HPeV, HRV | 3 |  |  |  |  |  |
|  | HBoV, HRV | 1 |  |  |  |  |  |
|  | HEV, HPeV | 1 |  |  |  |  |  |
|  | HEV, HRV | 1 |  |  |  |  |  |
|  | HEV, PIV | 1 |  |  |  |  |  |
|  | HRV, PIV | 1 |  |  |  |  |  |

Multiple viruses with ≥ 2 viruses in the nasopharyngeal sample.
